# Migration after union dissolution in the United States: The role of non-resident family

**DOI:** 10.1016/j.ssresearch.2021.102539

**Published:** 2021-02-13

**Authors:** Amy Spring, Clara H. Mulder, Michael J. Thomas, Thomas J. Cooke

**Affiliations:** a Department of Sociology, Georgia State University, 38 Peachtree Center Ave. SE, Atlanta, GA, 30302-5020, United States; b Population Research Centre, Faculty of Spatial Sciences, University of Groningen, PO Box 800, 9700 AV Groningen, the Netherlands; c Department of Geography, University of Connecticut, 215 Glenbrook Road, U-4148, Storrs, CT 06269, 4148, United States; d Statistics Norway, Akersveien 26, 0177 Oslo, Norway

**Keywords:** Migration, Union dissolution, Marital status, Social ties, Family networks, Return migration

## Abstract

Separation from a spouse or cohabiting partner is associated with a high likelihood of moving, even over long distances. In this paper, we use longitudinal data from the Panel Study of Income Dynamics for the United States to analyze the role of non-resident family in the migration of separated people immediately after and in the years following union dissolution. We explore both migration in general and return migration among separated people, drawing comparisons to married and never-married people. We find that having parents, children, or siblings living close by substantially deters migration, especially among separated people. We also find marked positive effects of having family members in the county where the respondent grew up on the likelihood of returning there. Separated people are especially likely to return, compared to others, if they have parents in their county of origin. Furthermore, a lack of an effect of years of education on migration, and a negative effect of this variable on return migration, suggest that migration after separation is less related to human-capital considerations than other types of migration.

## 1. Introduction

Separation – the dissolution of a co-residential union – is associated with a high likelihood of residential relocations (e.g. Clark 2013; Clark and Davies Withers 2007; Flowerdew and Al-Hamad 2004). For separated people, such relocations form an important instrument to establish a new household, and to adjust their housing situation and location to new needs and frequently a decrease in resources. A growing literature has addressed the residential relocations of separated people – whom we define as ex-partners after the dissolution of a marriage or a cohabiting partnership. Part of this literature has focused on the ‘event-move’ that at least one of the ex-partners makes to effectuate the separation (e.g. Gram-Hanssen and Bech-Danielsen, 2008; Mulder and Malmberg 2011; Mulder and Wagner 2010; Thomas et al., 2017). However, elevated mobility is also discernible when such ‘event-moves’ are disregarded (Feijten and Van Ham 2007). Furthermore, this elevated mobility extends several years beyond the event of separation (Feijten and Van Ham 2007, 2013; Kulu et al., 2021).

Many of the moves after separation cover only a short distance. In a less pronounced way, separation also seems to be associated with migration, that is, movement over longer distances (Clark and Davies Withers 2007 and Clark and Huang 2004 for the USA; Clark 2013, for moving 30+ km in Australia). Yet, only a few studies focus explicitly on the migration of separated people. Of those that do, most are restricted to migration immediately upon union dissolution (Cooke et al., 2016; Thomas et al., 2017; Mulder and Malmberg 2011).^1^

Long-distance movement of separated people is particularly relevant because it could open up new labor-market, social-support, and re-partnering opportunities for the separating person. At the same time, it could disrupt local social networks and harm the opportunities for contact with the non-resident parent for any involved children. Thus, for some separated people, there could be difficult tradeoffs between staying put and moving away. Arguably, the residential locations of non-resident family members could be crucial in these tradeoffs.

Researchers have studied long-distance movement in general (not specific to separated people) more extensively. It is often thought of as an economic endeavor – people who move long distances tend to do so for education and employment opportunities (Spring et al. 2016). Due to the prominence of economic explanations, the social aspects of long-distance migration have been relatively under-studied until more recently (Gillespie and Mulder 2020; Mulder 2018; Thomas and Dommermuth 2020). Recent studies suggest that for some people – depending on age, marital status, and other life circumstances – the social aspects may be particularly relevant for explaining migration decisions. These studies add nuance to understandings of long-distance moves, reconfiguring them as economic *and* social endeavors depending on life course considerations.

Social factors may be particularly important in the long-distance moves of separated people and for explaining their elevated rates of mobility. Researchers speculate that a considerable share of the moves of separated people might be return moves, for example, moves directed towards parents or other family members (Cooke et al., 2016). Yet, we know of no previous work devoted to return migration of separated people – except for a recent qualitative study showing that divorce was an important factor in return migration towards rural areas in the United States (Wall and Von Reichert 2013) and a study for the Netherlands in which some young adults mentioned divorce as a reason for return migration (Haartsen and Thissen 2014). At the same time, some studies are showing that divorce is associated with moving close to parents (Michielin et al., 2008; Smits 2010; Thomas and Dommermuth 2020) and moving in with them (Albertini et al., 2018; DaVanzo and Goldscheider 1990; Smits et al., 2010; Stone et al., 2014; Thomas and Dommermuth 2020). Furthermore, a study for the Netherlands by Das et al. (2017) showed that recently separated mothers of dependent children were more likely to move close to their mothers (the children’s maternal grandmothers), and to co-reside with them, than their counterparts who did not separate.

With this paper, we aim to contribute to the literature on residential relocations of separated people in three ways. First, we focus on long-distance moves not only immediately upon separation but also in the years after. Second, next to migration in general, we also address return migration of separated people. Third, we address the role of the residential locations of family members in separated people’s migration behavior, drawing comparisons to married and never-married people. We address the following research questions: How is separation associated with migration, and in particular, return migration? And, how is migration of separated people related to the residential locations of parents, children, and siblings – both at the current location and at the location of a potential return move? Using the Panel Study of Income Dynamics and multinomial logistic regression, we follow people for a period of 8 years from the time of union dissolution until the end of observation or re-partnering, and we compare the findings for separated people to married and never-married people. Our findings lend insight into who migrates and under what circumstances. We discuss the economic and social implications of these findings for separated people, given that social networks can be both a draw and constraint during the migration process.

## 2. Theoretical background

Our point of departure is an extended cost-benefit approach to the migration of separated people. Research on migration in the general population, or the population at working ages, frequently uses the classical cost-benefit approach. We extend this classical approach by also paying attention to costs and benefits associated with parents, siblings, and non-resident children living close by or (for return migration) in the region of birth. Return migration is usually seen as different from initial or onward migration; we therefore pay specific attention to it. We also pay attention to how migration after separation differs from migration in general, and formulate specific hypotheses for this type of migration. Some of these hypotheses compete with conventional hypotheses on migration.

### 2.1. An extended cost-benefit approach to migration

In the classical cost-benefit approach to migration, introduced by Sjaastad (1962), people are assumed to migrate if the benefits of a potential migration exceed the costs. From this perspective, younger people should migrate considerably more frequently than older people because of the longer time younger people have in front of them to recoup the costs of migration. Indeed, migration is strongly age-specific (Bernard et al., 2014). The main gains associated with migration are thought to be human-capital accumulation and increased returns on human capital. These gains are supposed to be higher for the highly educated. This explains why highly educated people are more likely to migrate than less educated people (Bernard and Bell 2018), and enrollment in education is associated with a high likelihood of migrating (Fischer and Malmberg 2001). Unemployment could also lead to increased benefits of migrating, whereas living in an urbanized area with many job opportunities could decrease these benefits.

The main costs of migration that have been put forward in classical cost-benefit approaches are non-monetary: the costs associated with severing ties to the location. Homeownership and children living in the household are local ties that are associated with a reduced likelihood of migration (Fischer and Malmberg 2001).

As Mulder (2018) suggested, it is useful to extend the classical cost-benefit approach to migration by introducing ties to non-resident family.^2^ Family members are important sources of social interaction and support exchange. Ties to non-resident family can therefore be viewed as a cost of migration if the family members live close by, or a benefit if they live far away. Indeed, as Clark et al. (2017), Thomas and Dommermuth (2020), and Mulder and Malmberg (2011, 2014) have shown, having family members living close by is an important local tie that decreases the likelihood of migration. Conversely, distant family members form an attraction factor for migration (Pettersson and Malmberg 2009; Smits 2010; Hedman 2013; Thomas and Dommermuth 2020).

The classical cost-benefit approach leads us to expect that the likelihood of migrating decreases with age, increases with education, and is greater for unemployed than employed people. Furthermore, those who own a home, have resident children, or live in urban areas would be less likely to migrate than others. Extending the cost-benefit approach leads us to expect that those who have family living close by will be less likely to migrate than those who do not. Furthermore, the likelihood of return migration would be enhanced if any family members live at the location of origin. We expect to find these family impacts in the general population too, but the magnitude of these impacts may be particularly high for separated people, a point to which we turn next.

### 2.2. Migration after union dissolution

Migration after union dissolution differs in several ways from any other migration. Separation itself is an important trigger for moving. As Feijten and Van Ham (2007) argued, moves triggered by separation tend to be urgent, financially restricted, and frequently also spatially restricted. They found that several categories of separated movers – particularly men with children and women without children – moved shorter distances than movers in partnerships. Because of the specificity of migration after separation, it could be that human-capital considerations play a less prominent part in it than in other types of migration. Rather than, or next to, being related to human capital, it might frequently be related to moving back to a previous location, for example to seek assistance (such as emergency housing, emotional support, or childcare support) from family or other social network members living at that location. A small-scale study by Wall and Von Reichert (2013) indeed suggested that divorce may be an important driver of return migration, and that “return migration to rural areas after divorce can be seen as a strategy to adapt to post-divorce changes and to cope with the trauma and vulnerabilities that can accompany divorce” (p. 350). Unlike what is usually found in the literature on migration in general, this would lead to a small effect of level of education, or perhaps the absence of such an effect. It would also lead to a strong negative effect of having family living close by on the likelihood of migration, and a particularly strong positive effect of family members living at the original location on the likelihood of returning there. For those with children, migration after separation is particularly complicated because they may need to remain close to the ex-partner to allow for contact with the children. Specifically for separated people, both resident children and children who live in close proximity could be a deterrent of migration. Given the potentially increased role of family networks in the migration of separated people, we derive the expectation that *having parents, siblings, or children living close by will be a stronger deterrent of migration for separated people compared to married or never-married people* (Hypothesis 1).

### 2.3. Return migration

The classical conceptualization of return migration is that it tends to be undertaken to recoup location-specific capital left at the original location, for example, to correct an initial move that was based on imperfect information and unsuccessful. Location-specific capital might include social capital, prior work experience, or knowledge of the local labor market. Following this idea, DaVanzo and Morrison (1981; see also DaVanzo 1983) found that return migrants in the U.S. tended to be less educated than onward migrants, and the unemployed were also particularly likely to return. Scholars have also suggested that return migration could be part of a sequence of moving towards and then returning from an ‘escalator region’ – a metropolitan region offering better opportunities for upward mobility than other regions. In such a sequence, successful migrants return to enjoy lower house prices and a better living environment (Fielding 1992; Champion 2012). Hunt’s (2004) findings for Germany suggested that return migrants may be a mixture of both successful and less successful migrants: A high level of education was positively associated with the likelihood of return migration in a similar way as other types of migration, but among men and younger people being laid off was particularly associated with return migration. A Swedish study of motives for migration revealed that, compared with non-return migration, people mainly undertook return migration for social reasons (Niedomysl and Amcoff 2011). Likewise, recent studies for the Netherlands (Zorlu and Kooiman 2019) and Sweden (Mulder et al., 2020) showed that return migration of young adults was much less likely if the parents no longer lived in the region of origin. Given the specific features of return migration, we anticipate that *separated people will be more likely to engage in return migration than married or never-married people* (Hypothesis 2), *particularly if they have family members living at the location of a potential return move* (Hypothesis 3).

### 2.4. The impact of gender

Gender differences may also arise from women’s generally greater economic and housing disadvantage after separation (Feijten, 2005). This disadvantage could lead women to be more likely to seek family assistance following separation than men. Women might also be more attached to or embedded in family networks. Studies have repeatedly shown that family relationships are stronger and family support is more frequent among women than men (Grigoryeva 2017; Rossi and Rossi 1990). Furthermore, because of women’s generally greater involvement in childrearing, the impact of having resident or non-resident children could also be greater. This leads us to expect that *family members’ locations will impact the migration of separated women more than the migration of separated men* (Hypothesis 4).

### 2.5. Education

If migration after separation is particularly motivated by social factors, an important question is what role, then, do human-capital considerations play? We have suggested that human-capital considerations, and especially education, might play a lesser role in migration after separation than in migration in general. Financial and social constraints following separation may mean that education, a very reliable predictor of migration in general, might be less related to migration after separation. But, findings on whether education is associated with an elevated likelihood of migration after separation are mixed. Cooke et al. (2016) found a positive association for newly separated people in the U.S., and Thomas et al. (2018) demonstrated that the geographical distance between separated parents was greater for highly educated than for less-educated parents in Great Britain. Findings, though, were different for Sweden. The distance of moves surrounding the event of separation in Sweden was not associated with education (Mulder and Malmberg, 2011). Researchers did not find an effect of education despite the fact that they used population data and thus statistical power was very large. Our analysis pays particular attention to education, with the hope of clarifying these mixed findings.

### 2.6. Locational disadvantage

Another driver of migration after union dissolution could be to undo a locational disadvantage. As postulated in the family migration literature (see Cooke, 2008, for an overview), couples and families are more restricted in choosing a residential location than single people. If a couple or family migrates, it is somewhat likely that one of the partners is a tied mover and ends up at a less-than-optimal location, or at least a location that is more advantageous to the partner than the individual. Cooke et al. (2016) have used the term ‘locational conflict’ to refer to this situation. As they argue, separation could be an occasion for the ex-partner who experiences such a locational conflict to solve this conflict by return migration. One would then expect the ex-partner who had less say in choosing a location to be more likely to migrate, particularly back to a familiar place. Such ex-partners would likely be those with less human capital than the other ex-partner: more likely the female than the male partner (in a two-sex couple), more likely those with less education and income than the ex-partner, and more likely the younger ex-partner. In line with these ideas, Cooke et al. (2016) found a noteworthy gender difference in the likelihood of interstate moves among newly separated people in the U.S. Separated women were considerably more likely to migrate than separated men, especially if they had contributed less to the total household income before the separation. Important for our study, experiencing a locational disadvantage may be associated with having had to move away from family members. People who move to less-than-optimal locations may therefore be particularly likely to have family members in the region of birth and to engage in return migration following separation. We explore the role of locational disadvantage in models for separated people that also assess the role of family locations.

Our analysis hopes to lend insight into the mixed findings of prior studies. By capturing a greater variety of familial relationships (parents, children, and siblings), following people for a longer period of time after union dissolution, and incorporating return migration, this study builds on prior studies in important ways to further our understanding of migration after separation. We discuss the broad implications of our findings for explaining the heightened mobility of individuals following separation, and for understanding the variable role of family networks in migration decisions.

## 3. Data and methods

### 3.1. Study design and data

We use data from the PSID, a nationally representative longitudinal survey of U.S. residents and their families, for the years 1983–2013. Members of the initial 1968 panel of approximately 5,000 families were interviewed annually until 1997 and biennially after that. New families have been added to the panel when children or other members of original panel families moved out to form their own households.

Panel attrition does not seem to be a big issue in the PSID. The PSID has maintained a re-interview rate between 95 and 98 percent across virtually all survey waves (PSID 2017) and assessments of the representativeness of the PSID find that income and health estimates align closely with other cross-sectional surveys (McGonagle et al., 2012; Schoeni et al., 2013). Yet, there might be some selective panel attrition among divorced individuals, which could have led to an underrepresentation of this category.

The multigenerational structure of the PSID allows us to track parents, children, and siblings living in separate households who are all related to an original sample family. We use the PSID supplemental Parent Identification file to link parents and children. Siblings are identified by linking respondents who have the same identifiers for one or more parents.

We categorize respondents as separated, currently married, or never-married. The currently married category also includes couples who the PSID determined were “permanently cohabiting”. Separation is defined as a transition out of the currently married/permanently cohabiting category, whether through a formal divorce or informal separation. We exclude respondents who are widowed from the analysis.

Separated respondents are followed from the time of divorce or separation until they re-partner or until the end of observation. Respondents who re-partner reenter the data as currently married. If they also separate from the new partner, they rejoin the separated category. We follow both former partners if information is available. However, in most cases information is only available for one of the former partners following union dissolution. The PSID continues to track only sample members or people living with sample members. Thus, if both former partners are PSID sample members (i.e., they were probably married in 1968), then both partners are tracked following union dissolution. Former partners who are non-sample members, but who live with one or more children from their former union (who are by definition sample members), are also tracked. In about 80% of cases; however, one former partner is not tracked because they are not sample members and do not reside with sample children following union dissolution. These tracking rules by the PSID are thought to be exogenous to migration decisions following divorce or separation. Our data track currently married and never-married respondents until the end of observation.

Data are structured as a series of person-intervals, each referring to the interval between successive interviews. We use the supplemental Geospatial Match files to link addresses of PSID respondents at each annual (or biennial) interview to corresponding codes for census tracts (PSID 2013). We then calculate distances between respondents’ census tracts from one interview to the next. Less than 1% of person-intervals are excluded from the analysis because they are missing geocodes. We also use the Geospatial Match files to link addresses of non-resident parents, children, and siblings at each interview to corresponding codes for census tracts; we then calculate distances between respondents and their non-resident kin. Locational information is only available for kin members who are also in the PSID sample and have non-missing geocodes; our data exclude other kin members. Despite these restrictions, we are able to link 98% of respondents to at least one non-resident family member in any given person-interval. We use the supplemental County Born/Grew Up files to link respondents to county identifiers for the location where they grew up. The county where they grew up is unknown for about 15% of respondents; we exclude these respondents from the analysis.

After removing those who are missing geocodes or county where grew up (and removing people who have been separated more than 8 years – a decision we describe below), the total sample of unique respondents is 19,551, including 3,246 separated respondents, 16,134 married respondents, and 5,523 respondents who have never been married.^3^ Separated respondents are followed for an average of 3.9 survey intervals after separation before they exit the data (because of re-partnering or the end of observation). Married and never-married respondents are followed for an average of 8.9 and 4.7 survey intervals, respectively. The final sample consists of 181,628 person-intervals for analysis, including 12,713 person-intervals for separated respondents, 143,144 person-intervals for married respondents, and 25,771 person-intervals for respondents who have never been married.

### 3.2. Measures

#### 3.2.1. Migration

A challenge of migration studies is selecting the threshold for the distance moved that qualifies as a “migration”. We rely on a mix of theory and previous research to select our threshold and then test the sensitivity of our results to our selection. Ultimately, we utilize 50 km (just over 30 miles) as the distance moved that qualifies as a migration. We chose 50 km because this distance is substantial enough to disrupt day-to-day activity spaces and local social and family networks, resulting in qualitatively different moves than localized moves. Also, a 50-km threshold is fairly consistent with other distance-based definitions of migration used in prior, comparable studies of family ties and migration (Ermisch and Mulder 2018). Relying on a distance-based measure over a boundary-based measure (for instance, defining migration as crossing state or county lines) has the added benefit of being more precise, since people could cross state or county boundaries while moving only a short distance.^4^

**Fig. 1.**
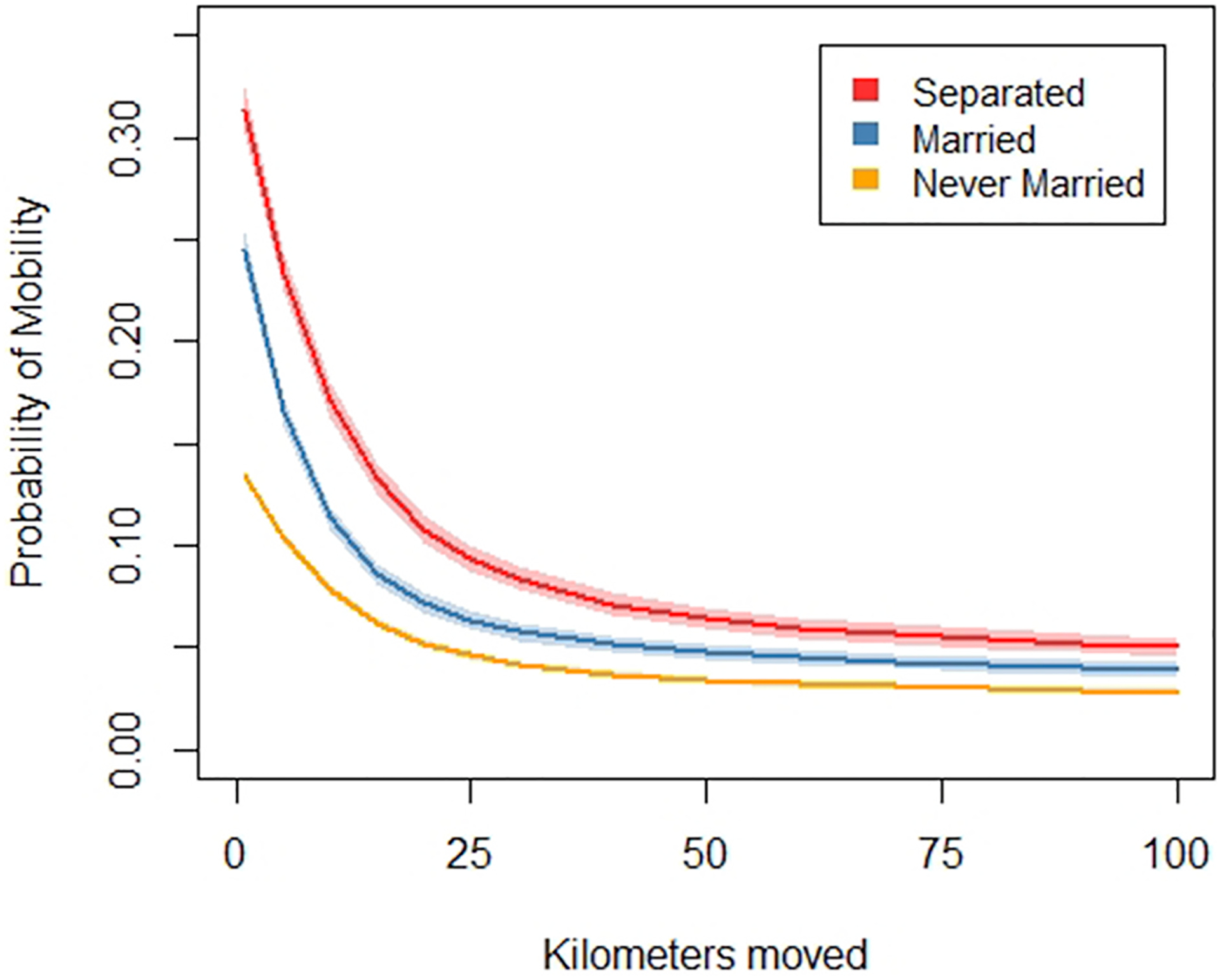
Marginal probability of mobility by distance moved by marital status.

**Fig. 2.**
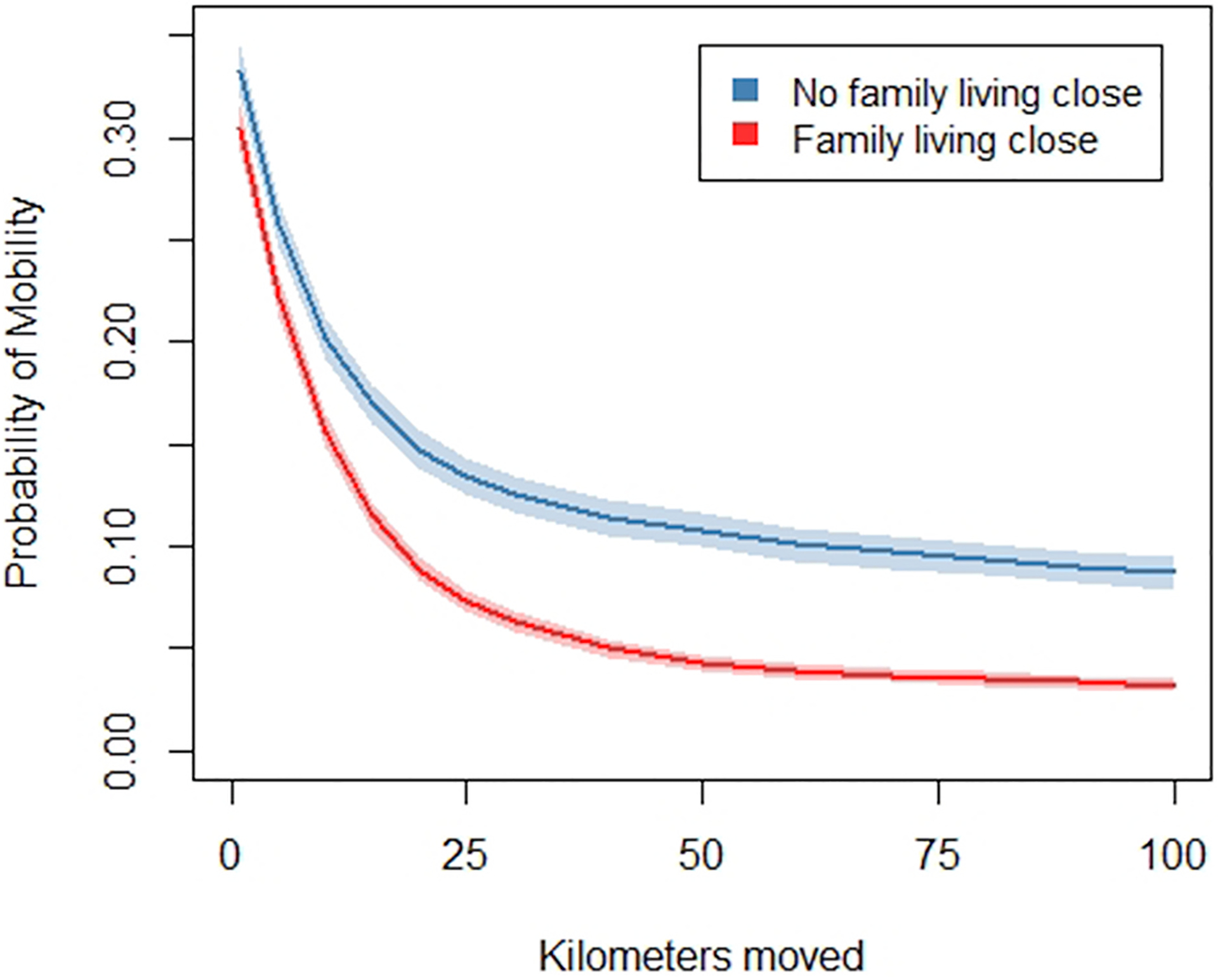
Marginal probability of mobility by distance moved by family living close at origins.

The choice of 50 km, as opposed to slightly more or less, is arbitrary. For that reason, we investigated the sensitivity of our results to the 50-km threshold. A mainstay of our findings is that migration rates are higher among separated people than married or never-married people. We find this to be true at all distances moved, from 1 km all the way to 100 km or more (Fig. 1). Another mainstay of our findings is that having family living nearby reduces the probability of migrating. Again, we find this to be true at all thresholds for distance moved, from 1 km all the way to 100 km or more (Fig. 2). These checks provide confidence that our results are not highly sensitive to our choice of a distance threshold.

Results are based on a series of logistic regressions predicting moves at each distance threshold adjusting for marital status, age, family income, and years of education. Robust standard errors are clustered by family identification numbers. The depicted marginal probabilities of moving hold age, family income, and years of education at their means. Shaded areas represent 95% confidence intervals.

Results are based on a series of logistic regressions predicting moves at each distance threshold, adjusting for whether family live close, marital status, age, family income, and years of education. Robust standard errors are clustered by family identification numbers. Depicted marginal mobility rates are for separated people with age, family income, and years of education held at their means. Shaded areas represent 95% confidence intervals.

**Fig. 3.**
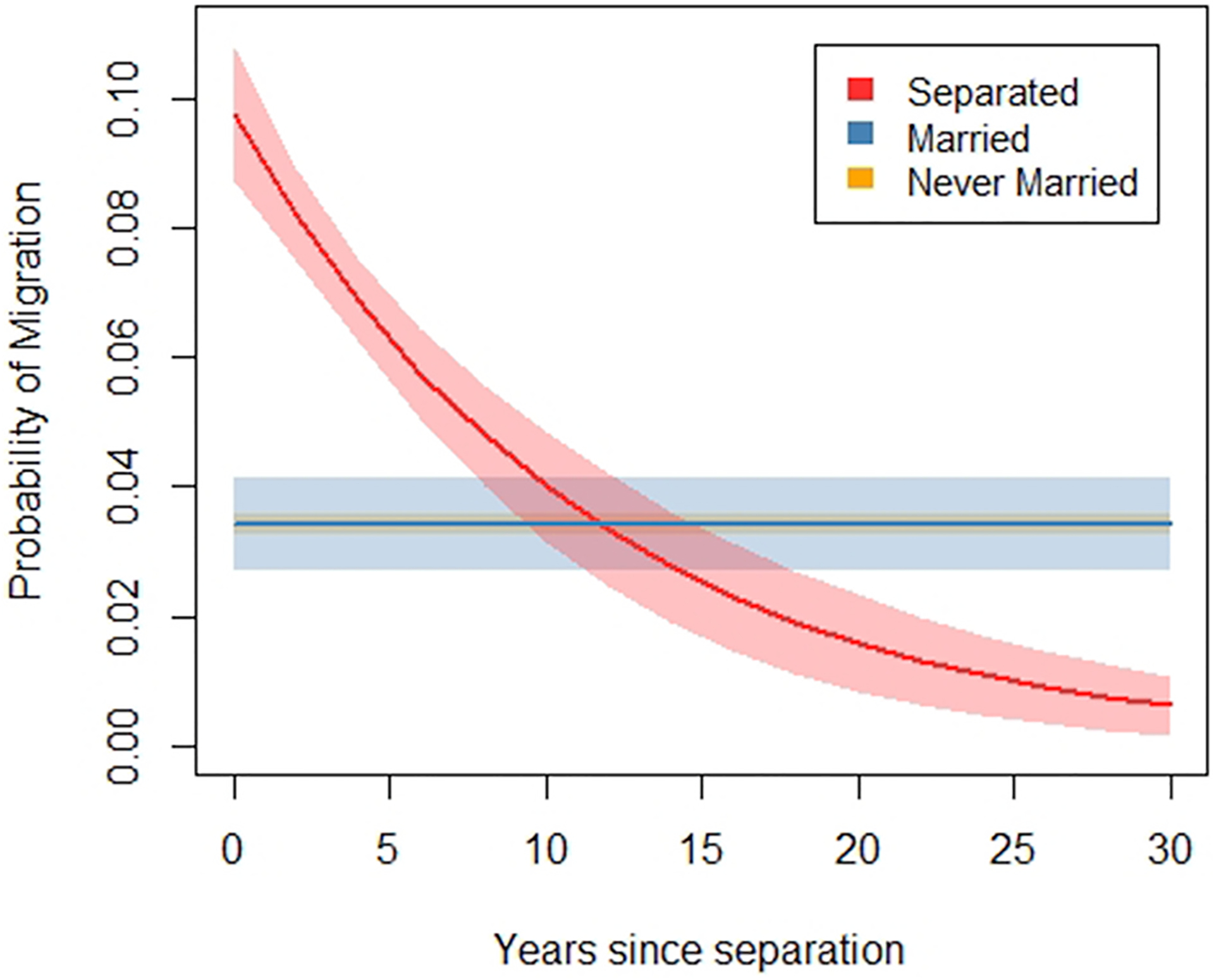
Marginal probability of migration by years since separation.

One innovation of our study over prior research is that we observe people at the time of separation and for a period after, allowing more time for migrations in response to separation to occur. A good question, though, is how long after separation can we still reasonably tie migration to the separation event? We considered limiting the window of time we observe people after separation, since some separated people remain in our data for one, two, or even three decades after the separation event. For guidance, we conducted a supplementary analysis of migration rates in the years after separation. Fig. 3 shows that migration rates for the separated peak at the time of separation (when years since separation = 0 on the graph), and then decline yet remain elevated above married and never-married people for about 8 years after separation. After 8 years, migration rates among the separated decline even further, below that of married and never-married people. Based on this information, we opted to limit the window of time we observe separated people to 8 years after separation. Future research should look into the potentially unique processes driving low rates of migration among those who have been separated for longer periods.

Results are based on logistic regressions predicting migration with age, family income, and years of education; separately for separated, married and never-married people. Robust standard errors are clustered by family identification numbers. In the model for separated people, years since separation is also included as a predictor variable. The depicted marginal probabilities of migration hold age, family income, and years of education at their means. Migration is defined as moving 50+km. Shaded areas represent 95% confidence intervals.

#### 3.2.2. Return migration

Another key contribution of our study is that we distinguish return migration from onward migration. Two criteria determine return migration in our study: 1) whether the respondent was currently living outside of the county where they grew up, and 2) whether by the next survey interval the respondent moved a distance of 50 km or more back to the county where they grew up. We code respondents meeting both of these criteria as return migrants. Respondents who moved shorter distances (i.e., less than 50 km) back to the county where they grew up are not coded as return migrants, since they moved only a short distance. Our definition of return migration is somewhat limited, since respondents may also return to other places they have previously lived or spent time. Thus, we are capturing a very specific type of return migration, which is back to the county where they grew up.

#### 3.2.3. Proximity to kin at origins

**Fig. 4.**
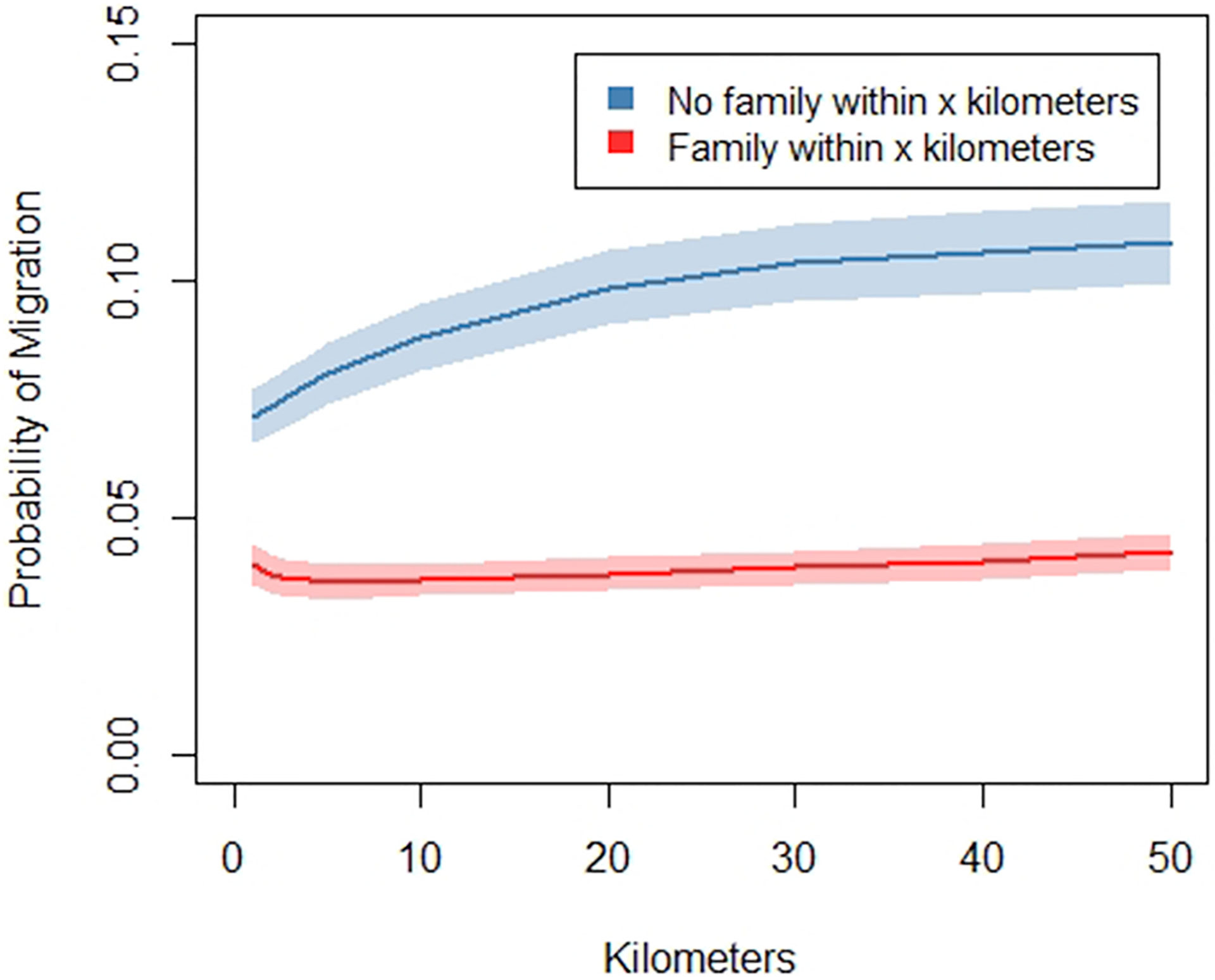
Marginal probability of migration by distance thresholds for family living “close” at origins.

Of interest to our study is whether having non-resident kin close by pre-migration influences whether a migration is made. We measure proximity to kin with dummy variables for whether one or more parents, siblings, or children live close to the respondent in the survey interval before a move. We again utilize the 50-km threshold to define which family members are living “close”. We wanted to be fairly inclusive of kin living nearby because even kin that are somewhat distant but still within the same city or metropolitan area could influence decisions to move far away. To capture all potentially theoretically-relevant kin, we ruled out distance thresholds that were much closer and, therefore, more restrictive. Also, as the distance cutoff got closer, we began to encounter sample size limitations for the number of people who had family living close and migrated. We conducted a supplemental analysis to examine whether our results change with different thresholds for living close. Fig. 4 shows that at all thresholds for living close, from 1 km up to 50 km, having family living close significantly reduces the likelihood of migration. These results suggest that our findings are not sensitive to our choice of a distance threshold for family living close. We also include control variables for whether the respondents’ parents live together – since migration away from or towards parents might be influenced by whether the parents are living together – and whether the respondent has no kin members identified in our data – since having no kin ties could influence migration.

#### 3.2.4. Resident kin

We also include measures of resident kin, defined as family members who live in the same family household as the respondent. We measure resident kin with dummy variables for whether the respondent has co-resident parents, siblings, or children. We combine parents and siblings into a single category due to small sample sizes. Resident children are divided by age into those who are 18 or older and those who are under 18 to account for migration differences that may arise from having minor versus adult children in the household. All measures of kin location are taken from the prior survey interview, before any potential move, with one exception. At the time of divorce or separation, we took the locations of children from the interview following the separation rather than the interview before, to account for the way custody arrangements might impact migration immediately following union dissolution.

Results are based on a series of logistic regressions predicting migration with marital status, age, family income, years of education, and varying distance thresholds for family living “close”. Robust standard errors are clustered by family identification numbers. Depicted marginal migration rates are for separated people with age, family income, and years of education held at their means. Migration is defined as moving 50+km. Shaded areas represent 95% confidence intervals.

#### 3.2.5. Kin in county where grew up

We include dummy variables for whether the respondent has parents, siblings, or children living in the county where they, the respondent, grew up. The locations of kin in the county where the respondent grew up are measured pre-move.

#### 3.2.6. Additional variables

We incorporate several additional variables that may impact migration decisions. They include the respondent’s sex, age, race/ethnicity (non-Latino white, non-Latino Black, or other race/ethnicity), education in years, employment status (employed, unemployed, or not in the labor force), and the study interval length (one year pre-1997 and two years after that). Household-level measures include whether the home is owned or rented, total family income from the previous year, region of the country, and size of the county’s largest city. Total family income is standardized to 2010 dollars and logged in regression models to account for substantial skewness. We include a control variable for the decade to account for temporal differences in migration and divorce rates. Finally, we include several variables for separated people that combine information from the respondent’s former partner to capture the concept of locational disadvantage. These variables include the difference in age between the respondent and their ex-partner (measured in years, with negative values indicating the respondent is younger and positive values indicating the respondent is older than their ex-partner), the difference in education (measured in years with negative values indicating the respondent had fewer years of education and positive values indicating the respondent had more years of education than their ex-partner), and the share of the couple’s income contributed by the respondent in the interview before divorce or separation (measured as a ratio ranging from 0 to 1 with 0.5 indicating that the respondent contributed income equal to their ex-partner). Up to 5.7% of values are missing for some variables; we impute missing values with multiple imputation.^5^

### 3.3. Analysis

We first present descriptive statistics on the migration rates, migration types, and sociodemographics of separated, married, and never-married respondents. We then present a series of multivariate models predicting migration and migration type. In all models, standard errors are clustered by family identification numbers to account for non-independence of observations of respondents within the same family, and multiple observations of the same respondents over time.^6^ In our first set of models, we use logistic regression to estimate the probability of migration for all people. We pay specific attention to how marital status and family locations independently and interactively predict migration. Our next models use multinomial logistic regression to predict migration type for all people. We estimate the probability of return migration for those currently living outside of the county where they grew up, as opposed to migrating elsewhere or not migrating. We again assess the independent impacts of marital status and family locations on migration type, and interactions between the two. Next, we re-estimate the same series of models for separated people only. In these models, we incorporate additional variables specific to separated people: years since separation, difference in age with their ex-partner, difference in education with their ex-partner, and share of income with their ex-partner. We assess whether relationships between migration and familial locations for separated people are robust to the additional variables. We also test for interactions between gender and family locations among separated people.

## 4. Results

### 4.1. Descriptive statistics

Table 1 shows the bivariate distributions of migration in our sample. In any given person-interval, 8.09% of separated people migrated. Of those, 2.04% returned to the county where they grew up, and 6.05% migrated elsewhere. Among married people, 3.75% migrated. Of those, 0.49% returned to the county where they grew up, and 3.26% migrated elsewhere. Among never-married people, 7.54% migrated. Of those, 1.74% returned to the county where they grew up, and 5.80% migrated elsewhere.

We report descriptive statistics of our independent variables in Table 2. Overall, nearly half of separated people had migrated before, meaning they were currently living outside of the county where they grew up, as opposed to 58.4% of married people and 40.8% of never-married people. A greater share of separated people lived near non-resident family members compared to married people. Separated people were also more likely than the married to have resident parents or siblings, and family members in the county where they grew up. On the other hand, never-married people were more likely than separated people to live close to family members and have family in the county where they grew up, with the exception of children. There are also marked differences by marital status in homeownership rates, age, income, race/ethnicity, and employment status, which collectively help explain differential rates of migration.

### 4.2. Analysis of migration by marital status

We begin the multivariate analysis by predicting migration in our full sample. The odds ratios reported in Table 3 demonstrate the elevated likelihood of migration among separated people. Compared to separated people, the odds of migration are 45% lower for married people and 44% lower for never-married people. The model demonstrates strong associations between having non-resident family members living within 50 km and the likelihood of migrating. In order of the magnitude of the effects, this is true of parents, children, and siblings. Having resident parents or siblings is also associated with a smaller likelihood of migrating. So is having a resident child, particularly if the child is younger than 18. Unsurprisingly, those who migrated before are more likely to migrate than those who did not.

The remainder of our independent variables operates as expected. The likelihood of migration declines with age and income and increases with education. Owning a home greatly reduces the odds of migration. Those who are male, white, and unemployed or not in the labor force are more likely to migrate, while those living in the Northeast and in large urban areas are less likely to migrate.^7^

To this model, we next add interactions between marital status and non-resident kin living close to test whether the location of kin is more impactful for the migration of separated people than for the migration of married or never-married people. To aid in the interpretation of results, we present figures showing the marginal predicted probability of migration, instead of the full regression table. The full results are available in Appendix Table A1. We graph the marginal predicted probabilities – which show the probability of migration for one category (e.g., a separated person without family nearby) compared to another (e.g., a separated person with family nearby) – with all other covariates held at their means. The graphs display bars representing 95% confidence intervals of the estimates to determine whether differences across categories are statistically significant.

**Fig. 5.**
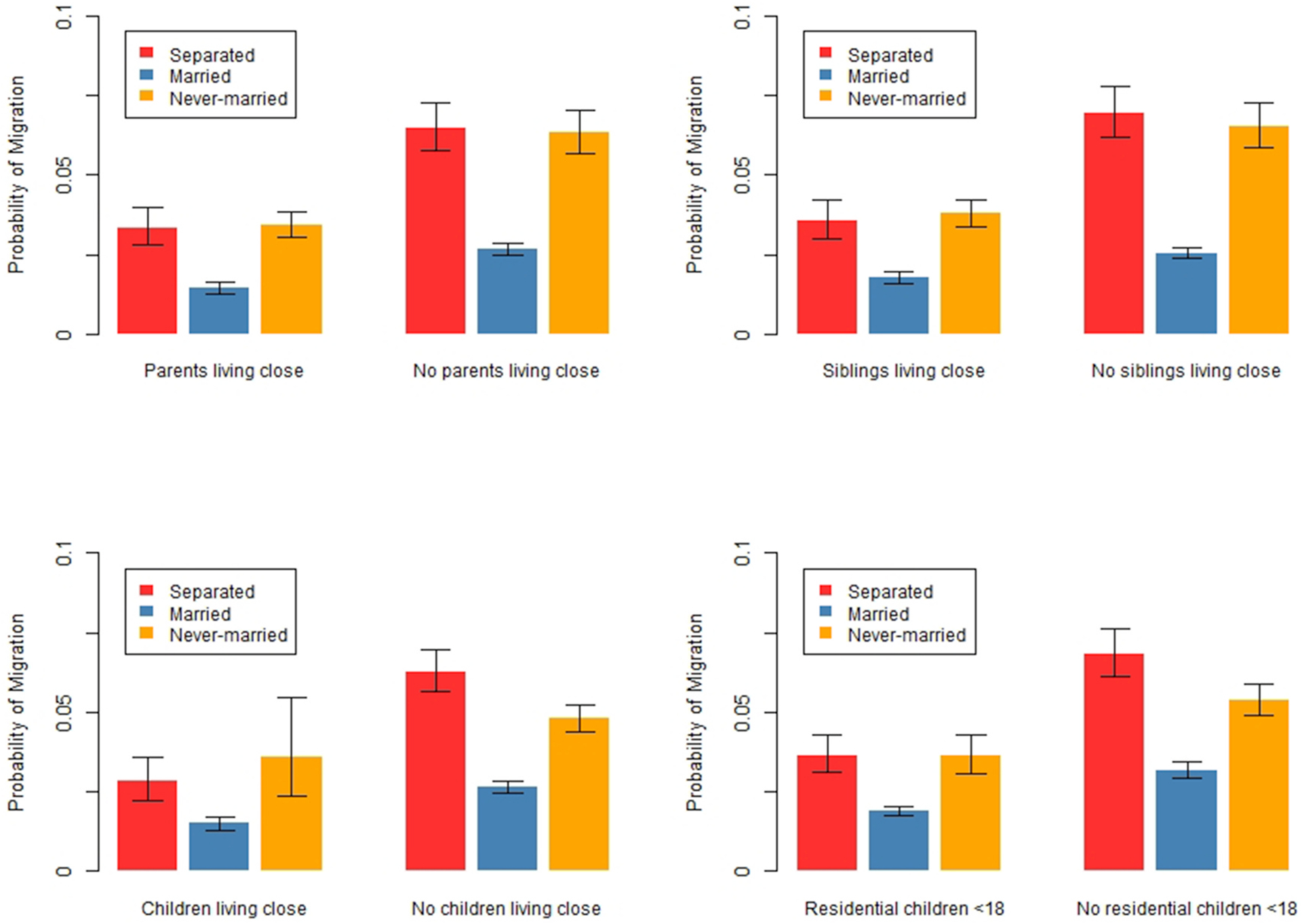
Marginal predicted probability of migration by marital status and family living close.

Fig. 5 shows the marginal predicted probability of migration by marital status for people with and without family living nearby. Having parents, siblings, or children living nearby significantly reduces the probability of migration for almost everyone. One exception is that having non-resident children nearby does not significantly reduce migration among never-married people, perhaps because too few never-married people have non-resident children to detect effects. Having resident children under 18 also significantly reduces the probability of migration for everyone. The magnitude of the reduction in the probability of migration from having family close is generally much greater for separated people than for married people, suggesting that family living close deters migration among separated people to a greater extent than married people. However, married people have a lower likelihood of migration than separated people, whether or not they have family close. The marginal effect of having family close is similar among separated and never-married people. Thus, Hypothesis 1 – that having parents, siblings, or children close by will be a stronger deterrent of migration for separated people – is supported when comparing separated and married people, but is not supported when comparing separated and never-married people. These results beg the question of whether it is being separated or simply being non-partnered that is most salient for tying people to locations near family. But, large standard errors for separated and never-married people make it difficult to disentangle these impacts.

Results are based on a logistic regression predicting migration with all independent variables from Table 3, plus interactions between marital status and family living close. Robust standard errors are clustered by family identification numbers. All other covariates are held at their means. Migration is defined as moving 50+km. Bars represent 95% confidence intervals. Full results are available in Appendix Table A1.

Separated people may be more likely to undertake specific types of migration, particularly migration back to the region where they grew up. Our next set of models investigate this possibility with multinomial logistic regression predicting return migration versus migrating elsewhere versus not migrating. This portion of the analysis is limited to people who have migrated before (i.e., they are currently living outside of the county where they grew up) since only these people are at risk of return migration. Table 4 reports the relative risk ratios for each type of migration. Results indicate that being married instead of separated decreases the risk of return migration versus not migrating by 71.9%, while being never-married instead of separated decreases the risk of return migration versus not migrating by 58.5%. In other words, separated people are more likely to engage in return migration than married or never-married people, lending support to Hypothesis 2. The likelihood of return migration is also substantially impacted by whether the respondent has family members living in the county where they grew up. In order of the magnitude of the effects, this is true of having children, parents, and siblings at the location of a potential return move. These findings align with other recent research (Mulder et al., 2020) and suggest that returning to the region of birth after separation is, to a large extent, a matter of returning to family.^8^ By comparison, the likelihood of migrating elsewhere versus not migrating is also greater for separated people than for married or never-married people. However, the magnitude of the differences by marital status is not as pronounced. Being married instead of separated decreases the risk of migrating elsewhere versus not migrating by 41%, and never being married instead of separated decreases the risk of migrating elsewhere versus not migrating by 46.8%. Having family in the county where they grew up has no bearing on respondents’ decisions to migrate elsewhere versus not migrating.

Among the other independent variables, return migration (versus not migrating) is less likely among women, among people who have family members living close by pre-move, and among people with resident children under 18. Migrating elsewhere is also less likely among these groups; however, having family living close by pre-move appears to deter return migration more than it deters migrating elsewhere. And having resident parents, siblings, or adult children, which does not impact return migration, does deter migrating elsewhere.^9^

The likelihood of return migration (or migrating elsewhere) versus not migrating declines with age, owning a home, being employed, and living in a large urban area. Income and education, on the other hand, show differential effects on return migration and migrating elsewhere. Higher income reduces the likelihood of return migration but has no impact on migrating elsewhere. In contrast, greater education increases the likelihood of migrating elsewhere but has no impact on return migration. Race/ethnicity also appears to have differential impacts on migration, depending on the type. Whites are somewhat more likely to engage in return migration than Blacks, but much more likely to engage in migration elsewhere than Blacks. These results suggest that return migration is indeed specific. Whereas migration in general is invariably positively related to education and frequently related to job changes or enrollment in education, return migration is likely more related to seeking support, shelter or company from family, and/or returning to a familiar environment where location-specific capital has been left behind.

To this model, we next add interactions between marital status and having family members in the county where the respondent grew up. We include these interactions to examine Hypothesis 3 - that separated people are more likely than married or never-married people to engage in return migration if they have family members living at the location of a potential return move. We present figures showing the marginal predicted probability of migration, focusing only on return migration. The full regression results are available in Appendix Table A2.

**Fig. 6.**
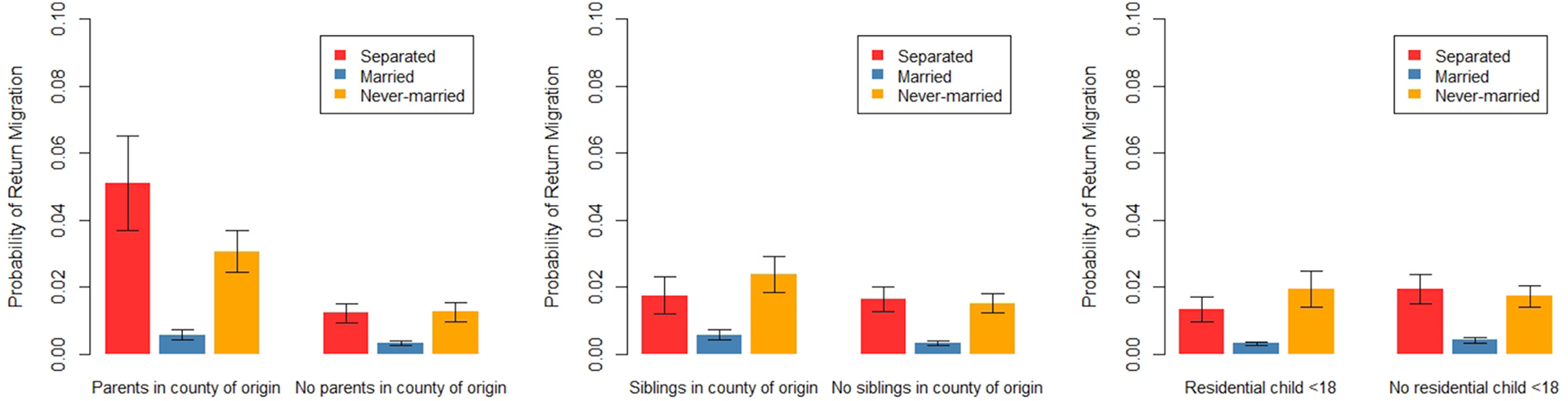
Marginal predicted probability of return migration by marital status and family living in the county where the respondent grew up.

Fig. 6 shows the marginal predicted probability of return migration (versus not migrating) by marital status for people with and without family living in the county where they grew up. All other covariates are held at their means. Results indicate that having parents in the county where respondents grew up significantly increases the likelihood of return migration across all marital statuses – but especially for separated people. In contrast, having siblings in the county where one grew up significantly increases the likelihood of a return migration for married people only. And despite the increase from having siblings, married people still have very low rates of return migration compared to other groups. Having resident children under 18 does not differentially impact return migration across marital status. Sample sizes were too small to estimate interactions between marital status and having children in the county where the respondent grew up – and for that reason, those results are not reported. In sum, the interactions lend partial support to Hypothesis 3. Having parents (but not siblings) in the county where they grew up increases the likelihood of return migration among separated people more than it does married or never-married people. Furthermore, having resident children under 18 does not impact return migration more for separated people than married or never-married people.

Results are based on a multinomial logistic regression predicting return migration versus not migrating with all independent variables from Table 4, plus interactions between marital status and family living in the county where the respondent grew up. Robust standard errors are clustered by family identification numbers. All other covariates are held at their means. Return migration is defined as moving 50+km back to the county where the respondent grew up. Bars represent 95% confidence intervals. Full results are available in Appendix Table A2.

### 4.3. Analysis of migration for separated people only

Next, we turn to multivariate models of migration for separated people only. Focusing on separated people allows us to incorporate several additional variables that may impact the migration of separated people in particular, and may help clarify relationships between the migration of separated people and the location of family members. These additional variables include years since separation, and three potential indicators of locational disadvantage: the difference in age between the respondent and their ex-partner, the difference in education between the respondent and their ex-partner, and the ratio of the household income contributed by the respondent before the separation.

Table 5 shows that among the separated, the likelihood of migration is still significantly reduced for people who have family living close by, despite the inclusion of the additional variables. In other words, relationships between migration and family living close are robust to other explanations for migration among separated people, particularly, the number of years they have been separated and whether they were the partner most likely to have experienced a locational disadvantage. Results for the new variables indicate that each additional year since separation reduces the odds of migrating by 14% (for up to 8 years post-separation since that is where we censor our sample). Differences in age, education, or the share of income with the ex-partner do not seem to impact the likelihood of migration. Given these results, we do not find evidence that the ex-partner who had less say in choosing a location is more likely to migrate.

Among the other variables’ coefficients, the lack of an effect of education is noteworthy. This result is in line with the idea that human-capital considerations are less important to migration after separation than to other types of migration. The finding is similar to that of Mulder and Malmberg (2011) for migration upon separation in Sweden, but different from studies that found positive effects of level of education on migration after separation (Cooke et al., 2016, for the USA and Thomas et al., 2018, for Great Britain). However, our sensitivity analyses of the distance threshold for moving indicate that education’s effect on migration after separation increases with the distance of the move. Highly educated people are more likely than less-educated people to move 200 km or more following separation, whereas for shorter moves, the difference is not significant. These results do still point to a variable role of education in migration following separation, in line with prior mixed findings. But, these results also lend support to the idea that education plays a more complex, inconsistent, and less prominent role in migration after separation than in migration in general.

**Fig. 7.**
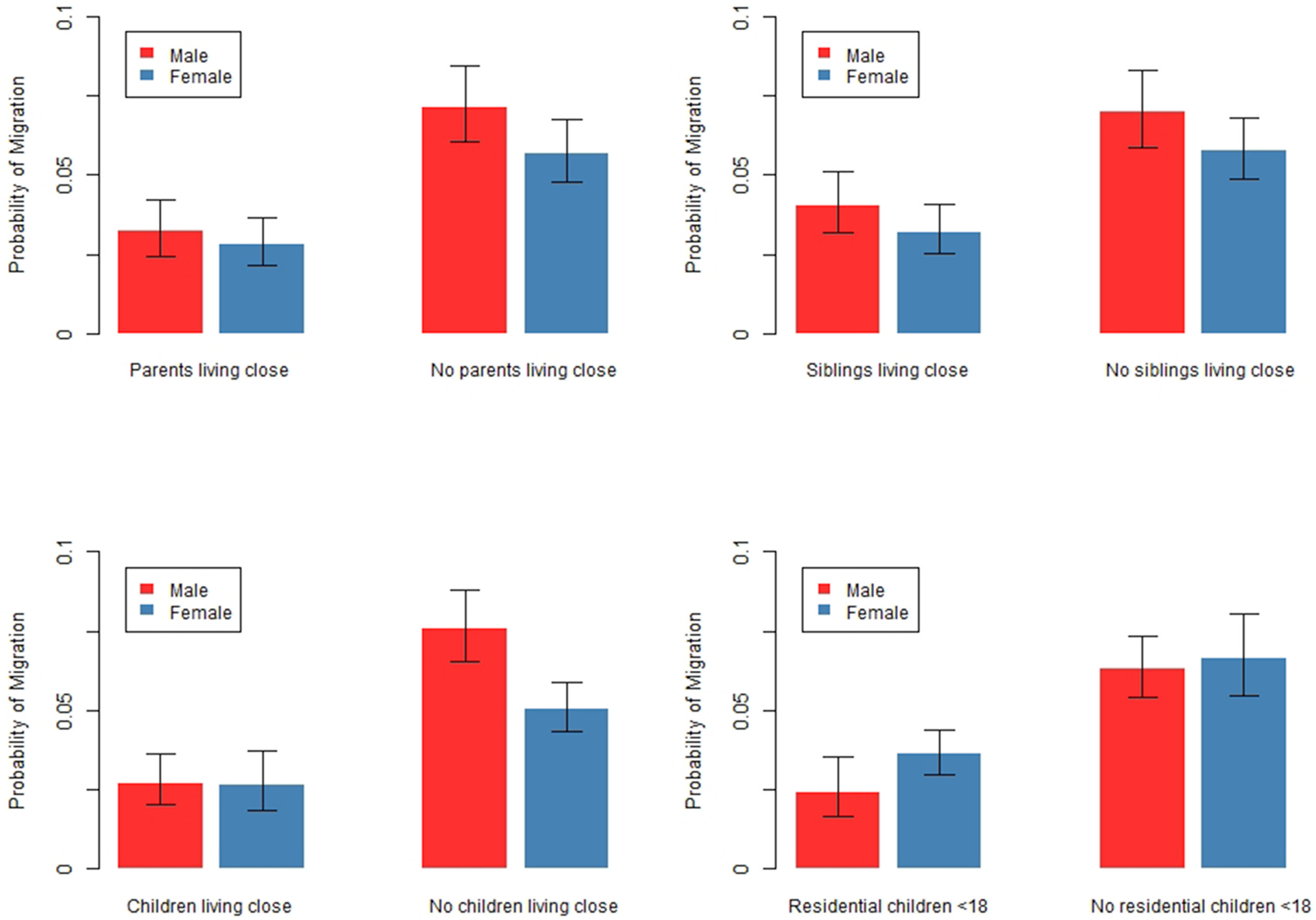
Marginal predicted probability of migration among separated people by sex and family living close.

Among the separated, we also examine the possibility that the location of family members impacts migration more for women than for men. To test this idea, we add interactions between sex and family locations to the model. We report the marginal predicted probabilities of migration, by sex and family locations, with all other covariates held at their means. Fig. 7 shows that having family close by impacts migration similarly among separated men and separated women. The full results (available in Appendix Table A3) indeed confirm the lack of a significant interaction between sex and family locations. These results lend no support to Hypothesis 4, which stated that the location of family members would impact the migration of separated women more than separated men.

Results are based on a logistic regression predicting migration among separated people with all independent variables from Table 5, plus interactions between sex and family living close. Robust standard errors are clustered by family identification numbers. All other covariates are held at their means. Migration is defined as moving 50+km. Bars represent 95% confidence intervals. Full results are available in Appendix Table A3.

Lastly, we turn to an analysis of migration type for separated people only. We report the results of a multinomial logistic regression predicting return migration versus migrating elsewhere versus not migrating among separated people. Again, we add years since separation, and the difference in age, education, and share of income with the ex-partner to the model. Table 6 shows that having parents and children in the county where they grew up significantly increases the likelihood of return migration among the separated. Having siblings in the county where separated people grew up does not impact the odds of return migration, as siblings do in the full sample based on the results from Table 4. This confirms the lack of an effect for siblings among separated people, which we also saw in the interactions reported in Fig. 6. Moreover, relationships between family locations and return migration for separated people appear robust to other considerations, including years since separation and indicators of locational disadvantage. Results indicate that each additional year since separation decreases the risk of return migration versus not migrating by 22%, and decreases the risk of migrating elsewhere versus not migrating by 12.2%. Differences in age with the ex-partner influence return migration but not migrating elsewhere, and not in the expected direction. The respondents’ risk of return migration (versus not migrating) increases by 3.3% for each additional year older they are than their ex-partner. Differences in education and share of income with the ex-partner do not seem to impact migrations.

**Fig. 8.**
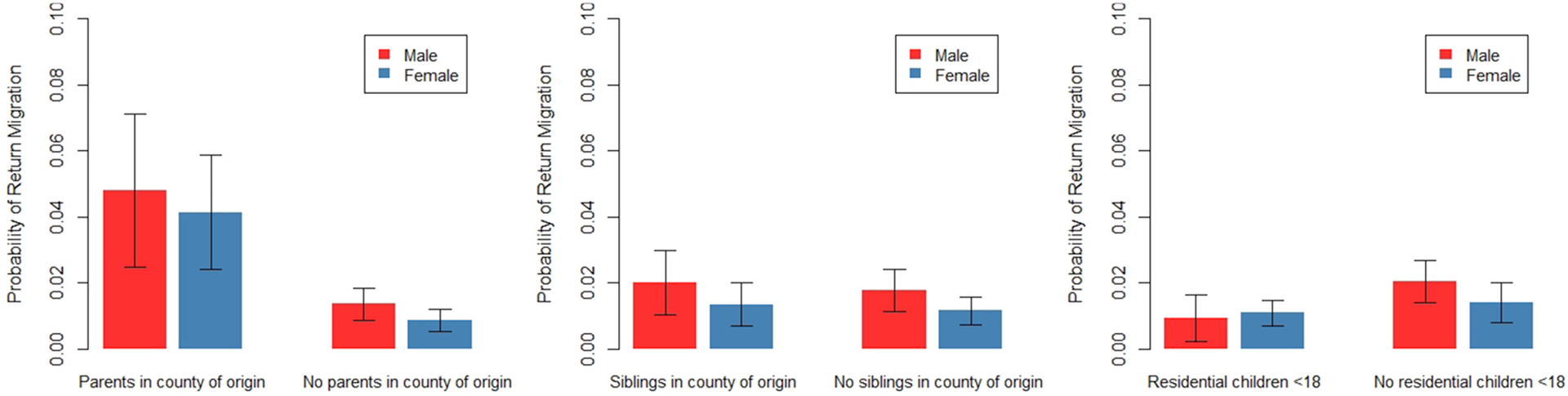
Marginal predicted probability of return migration among separated people by sex and family living in the county where the respondent grew up.

To this model, we add interactions between sex and family living in the county where the respondent grew up. We report the marginal predicted probability of return migration among separated people in Fig. 8. Results are reported separately for males and females, and for people with and without family living in the county where they grew up. All other covariates are held at their means. The full table of results is available in Appendix Table A4. We find no significant interactions between sex and family locations when predicting return migration among the separated. In other words, the location of family members in the county of origin seems to impact return migration similarly among separated men and women.

The lack of gender differences in our study contradicts previous findings from Cooke et al. (2016). They also used the PSID and found that separated women were more likely to migrate than separated men. We performed additional analyses to find out what causes this discrepancy in findings based on the same data. We found that the differing definition of migration was the main cause of differences in our findings: we use a distance threshold, whereas Cooke and colleagues used interstate moves. As Cooke and colleagues argue based on Nazir (2009), state laws and state court decisions may restrict interstate moves following union dissolution. State boundaries may form a greater barrier for separated men than separated women, a point which further studies should follow up on.

Results are based on a multinomial logistic regression predicting return migration versus not migrating among separated people with all independent variables from Table 6, plus interactions between sex and family living in the county where the respondent grew up. Robust standard errors are clustered by family identification numbers. All other covariates are held at their means. Return migration is defined as moving 50+km back to the county where the respondent grew up. Bars represent 95% confidence intervals. Full results are available in Appendix Table A4.

## 5. Conclusion and discussion

In this paper, we addressed the role of family in migration after separation in the United States. We hypothesized that having non-resident family living close by would deter the migration of everyone, but especially separated people. We further hypothesized that separated people would be more likely than others to return to the county where they were grew up, especially if they had family there. We found support for each of these hypotheses to some extent. Our findings indicated that non-resident parents, siblings, and children play a decisive part in the likelihood and direction of migration after separation. The effect of having a parent in the county of origin on return migration was particularly large. If parents no longer live in the region of birth, this region apparently loses much of its attractiveness as a place to return. We also hypothesized a greater role of family in women’s than in men’s migration after separation, but our findings did not support this expectation.

We found further evidence that migration of separated people, and particularly return migration, differs from other types of migration. In contrast to findings for migration in general, no effect of education was found on migration of separated people – at least not for the distance threshold we used –, and for return migration the effect was negative. This difference in findings suggests that the migration of separated people is less related to human capital than other types of migration. In principle, this could also mean that migration after separation could be a way to undo a locational disadvantage. Yet, in contrast with Cooke et al. (2016), we did not find support for the idea that those who were likely to have had less say in previous migration decisions and thus more likely to experience a locational disadvantage (i.e., women, and those with less education and income than their partner) would be more likely to migrate than others. The differences between our findings and theirs may be related to different definitions of migration: Whereas they analyzed interstate moves, we used a distance threshold. Although sensitivity analyses using different distance thresholds (including our own) usually do not lead to very different results, U.S. state boundaries may be specific because of jurisdiction regarding parents’ rights after divorce. Future research should explore how distance combines with crossing state or county boundaries to influence the migration of separated people, particularly separated people with children.

Our study contributes to the literature on internal migration in two ways. First, it emphasizes once more the importance of non-resident family in migration, both as a deterrent (if family members live close by) and as an attraction factor (if they live at a longer distance). Second, it demonstrates the importance of distinguishing between types of migration and studying these separately: migration after separation versus moves in the general population; and return migration versus migration elsewhere. To the literature on separation and divorce, our study contributes knowledge about how separated people choose their residential locations, with attempts to maintain or seek closer proximity to family members clearly an important factor in post-separation recovery processes.

Naturally, our study has limitations. Our measure of return migration is somewhat simplistic: We study returns to the county where respondents grew up and disregard moves to other areas where people may have spent time or have family networks. And although we include measures of family in the county of origin, it is still unclear what return movers are returning to. In addition to family members, return migration may provide access to fictive kin, friends, and familiar cultures and locations. Further research describing motivations for return migration could shed light on why separated people are especially likely to undertake this type of migration. Furthermore, although the PSID sample is large, the numbers are not large enough to allow even more detailed analyses such as moving to live close to a parent or moving in with a parent after separation. The only feasible option to perform such analyses are data from combinations of administrative registers or population registers and census data that include parent-child links and residential locations. Such data are now available for several European countries (for example, Norway, Sweden, Finland, Denmark, the Netherlands, and Belgium).

Our study highlights the important role of non-resident family in migration after separation. More broadly, our results emphasize the social drivers of migration, which may be particularly relevant for certain groups or particular life transitions. Loss of a job, death of a spouse, home foreclosure, health problems, and other adverse life events and circumstances might cause the same types of moves with a similar role of non-resident family. Future work could focus on migration after such events or in such circumstances to help clarify and extend theories of migration. Like our current analysis of migration after separation, all of these focused analyses provide insight into responses to events that increase people’s vulnerability and would be particularly interesting for policy makers.

## Figures and Tables

**Table 1:** Migration distributions by marital status.

	Separated	Married	Never-married
	%	%	%
Migrated	8.09	3.75	7.54
Migration Type			
Returned to county of birth	2.04	0.49	1.74
Migrated elsewhere	6.05	3.26	5.80
Did not migrate	91.91	96.24	92.46
N (person-intervals)	12,713	143,139	25,761

**Table 2:** Descriptive statistics.

	Separated		Married		Never-married	
	Mean	(SD)	Mean	(SD)	Mean	(SD)
Migrated before	.489		.584		.408	
Non-resident parent within 50 km	.341		.203		.479	
Non-resident sibling within 50 km	.442		.224		.596	
Non-resident child within 50 km	.243		.218		.065	
Parents living together	.230		.160		.279	
No non-resident kin in PSID	.019		.045		.020	
Resident parents or siblings	.118		.020		.188	
Resident child < 18	.444		.578		.328	
Resident child 18+	.132		.184		.067	
Parent in county where grew up	.322		.216		.480	
Sibling in county where grew up	.389		.216		.538	
Child in county where grew up	.117		.100		.045	
Female	.589		.473		.608	
Homeowner	.415		.759		.177	
Age	38.415	(10.580)	42.865	(13.577)	31.593	(9.912)
Total family income ($1,000s)	41.716	(38.466)	68.957	(76.068)	24.919	(32.127)
Education in years	12.715	(2.281)	12.901	(2.709)	12.783	(2.265)
Study interval length	1.383	(.486)	1.340	(.474)	1.413	(.492)
Race/Ethnicity						
White, Non-Latino	.537		.693		.374	
Black, Non-Latino	.396		.225		.578	
Other race/ethnicity	.067		.082		.048	
Year						
1983 to 1989	.217		.302		.255	
1990 to 1999	.449		.396		.367	
2001 to 2013	.335		.302		.378	
Region						
Northeast	.113		.161		.133	
Midwest	.215		.249		.249	
South	.498		.412		.466	
West	.173		.178		.152	
Employment status						
Employed	.754		.724		.699	
Unemployed	.092		.404		.148	
Not in labor force	.154		.235		.153	
Size of largest city in county						
<25,000	.337		.363		.212	
25,000–99,999	.216		.233		.201	
100,000 or more	.448		.404		.587	
Years since separation	2.488	(2.426)				
Difference in age w/ex-partner	−.226	(5.741)				
Difference in education w/ex-partner	.119	(2.228)				
Share of income w/ex-partner	.487	(.316)				
N (person-intervals)	12,713		143,139		25,761	

Note: Values are for non-missing observations.

**Table 3:** Logistic regression of migration (ref. did not migrate).

	OR	SE
Marital status (ref = separated)		
Married	.549***	.027
Never-married	.562***	.033
Migrated before	2.264***	.079
Female	.888***	.022
Non-resident parent within 50 km	.545***	.023
Non-resident sibling within 50 km	.623***	.025
Non-resident child within 50 km	.552***	.035
No non-resident kin in PSID data	.645***	.042
Parents living together	1.134**	.046
Resident parent or sibling	.742***	.044
Resident child < 18	.595***	.025
Resident child 18+	.657***	.044
Age	.963***	.002
Race (ref = non-Latino white)		
Non-Latino black	.585***	.033
Other	.526***	.050
Homeowner	.344***	.014
Total family income (logged)	.956**	.016
Education in years	1.082***	.010
Employment (ref = employed)		
Unemployed	1.544***	.075
Not in labor force	1.817***	.070
Region (ref = Northeast)		
Midwest	1.225**	.088
South	1.419***	.092
West	1.673***	.122
Size of largest city in county (ref=<25,000)		
25,000–99,999	1.062	.050
100,000 or more	.840***	.038
Year (ref = 1983 to 1989)		
1990 to 1999	1.047	.045
2001 to 2013	.954	.073
Study interval length	1.833***	.121
Constant	.103***	.019
N (person-intervals)	181,628	

* p < .05,

** p < .01,

*** p < .001.

Note: Pooled results of 10 imputation datasets; standard errors are clustered by family identification number.

**Table 4:** Multinomial logistic regression of migration type for those who migrated before (ref. did not migrate) (N = 100,400).

	Returned to county where grew up		Migrated elsewhere		
	RRR	SE	RRR		SE
Marital status (ref = separated)					
Married	.281***	.025	.590	***	.039
Never-married	.415***	.044	.532	***	.041
Female	.851*	.054	.885	***	.028
Parent in county where grew up	2.490***	.228	.908		.053
Sibling in county where grew up	1.478***	.124	1.010		.056
Child in county where grew up	4.264***	.713	1.220		.208
Non-resident parent within 50 km	.205***	.033	.566	***	.036
Non-resident sibling within 50 km	.394***	.048	.583	***	.036
Non-resident child within 50 km	.531***	.078	.493	***	.039
No non-resident kin in PSID data	1.167	.186	.661	***	.055
Parents living together	.980	.077	1.074		.061
Resident parent or sibling	.792	.108	.754	**	.074
Resident child < 18	.805*	.068	.640	***	.032
Resident child 18+	.816	.137	.651	***	.051
Age	.942***	.006	.969	***	.002
Race (ref = non-Latino white)					
Non-Latino black	.833*	.076	.538	***	.038
Other	.473***	.079	.508	***	.058
Homeowner	.311***	.026	.333	***	.017
Total family income (logged)	.906***	.025	.978		.023
Education in years	.974	.014	1.089	***	.012
Employment (ref = employed)					
Unemployed	1.829***	.181	1.442	***	.098
Not in labor force	1.900***	.160	1.795	***	.089
Size of largest city in county (ref=<25,000)					
25,000–99,999	1.086	.099	1.025		.057
100,000 or more	.784**	.066	.838	**	.054
Region (ref = Northeast)					
Midwest	1.153	.145	1.170		.103
South	1.558***	.175	1.406	***	.112
West	1.582***	.199	1.600	***	.143
Year (ref = 1983 to 1989)					
1990 to 1999	.968	.083	1.122	*	.061
2001 to 2013	1.030	.178	.928		.087
Study interval length	1.843***	.284	1.921	***	.153
Constant	.452*	.165	.115	***	.026
N (person-intervals)	1,411		5,001		

* p < .05,

** p < .01,

*** p < .001.

Note: Pooled results of 10 imputation datasets; standard errors are clustered by family identification number.

**Table 5:** Logistic regression of migration (ref. did not migrate) for separated people only.

	OR	SE
Migrated before	2.737***	.241
Female	1.046	.115
Non-resident parent within 50 km	.472***	.050
Non-resident sibling within 50 km	.571***	.056
Non-resident child within 50 km	.418***	.050
No non-resident kin in PSID data	.396*	.150
Parents living together	1.076	.110
Resident parent or sibling	.562***	.082
Resident child <18	.486***	.049
Resident child 18+	.663**	.098
Age	.965***	.005
Race (ref = non-Latino white)		
Non-Latino black	.603***	.068
Other	.672	.141
Homeowner	.469***	.040
Total family income (logged)	1.023	.048
Education in years	1.013	.021
Employment (ref = employed)		
Unemployed	2.076***	.239
Not in labor force	1.904***	.206
Region (ref = Northeast)		
Midwest	1.581*	.280
South	1.753**	.288
West	1.997***	.342
Size of largest city in county (ref=<25,000)		
25,000–99,999	.906	.094
100,000 or more	.663***	.065
Year (ref = 1983 to 1989)		
1990 to 1999	1.096	.115
2001 to 2013	1.162	.224
Study interval length	1.445*	.235
Years since separation	.860***	.018
Difference in age with ex-partner	1.008	.008
Difference in education with ex-partner	.983	.020
Ratio of income with ex-partner	1.048	.158
Constant	.239***	.094
N (person-intervals)	12,713	

* p < .05,

** p < .01,

*** p < .001.

Note: Pooled results of 10 imputation datasets; standard errors are clustered by family identification number.

**Table 6:** Multinomial logistic regression of migration type for those who migrated before for separated people only (ref. did not migrate) (N = 6,218).

	Returned to county where grew up		Migrated elsewhere	
	RRR	SE	RRR	SE
Female	.923	.178	1.067	.143
Parent in county where grew up	4.966***	1.009	.615**	.107
Sibling in county where grew up	1.179	.215	1.227	.201
Child in county where grew up	3.075***	.962	1.253	.309
Non-resident parent within 50 km	.154***	.047	.517***	.083
Non-resident sibling within 50 km	.438***	.100	.562***	.087
Non-resident child within 50 km	.557**	.119	.418***	.063
No non-resident kin in PSID data	.248	.256	.573	.269
Parents living together	.891	.162	1.051	.154
Resident parent or sibling	.670	.236	.543*	.136
Resident child < 18	.734	.135	.488***	.063
Resident child 18+	1.247	.332	.581**	.105
Age	.950***	.010	.972***	.006
Race (ref = non-Latino white)				
Non-Latino black	.719	.144	.524***	.084
Other	.364**	.139	.808	.240
Homeowner	.442***	.076	.490***	.056
Total family income (logged)	.960	.072	1.106	.075
Education in years	.911*	.033	1.033	.029
Employment (ref = employed)				
Unemployed	2.504***	.562	2.323***	.374
Not in labor force	2.378***	.463	2.056***	.315
Size of largest city in county (ref=<25,000)				
25,000–99,999	1.109	.207	.820	.114
100,000 or more	.719	.131	.605***	.083
Region (ref = Northeast)				
Midwest	1.716	.526	1.406	.347
South	2.052*	.590	1.507	.351
West	1.684	.537	1.847**	.433
Year (ref = 1983 to 1989)				
1990 to 1999	.993	.196	.985	.141
2001 to 2013	.688	.259	1.125	.306
Study interval length	2.293**	.710	1.339	.322
Years since separation	.781***	.036	.878***	.025
Difference in age with ex-partner	1.033*	.015	1.004	.009
Difference in education with ex-partner	1.021	.041	.948	.028
Ratio of income with ex-partner	.783	.217	1.060	.211
Constant	.575	.417	.269*	.140
N (person-intervals)	259		537	

* p < .05,

** p < .01,

*** p < .001.

Note: Pooled results of 10 imputation datasets; standard errors are clustered by family identification number.

## Footnotes

1 While not focusing on migration, Thomas et al. (2018) performed a longitudinal analysis of distances between ex-partners after separation.

2 Others have proposed a different extension: including consumer amenities, cultural attractions and nice weather (see Niedomysl and Clark, 2014 for an overview).

3 The numbers of separated, married, and never-married respondents do not sum to the reported total of unique respondents because the marital status categories are not mutually exclusive.

4 However, crossing state or county lines may be particularly salient predictors of moving for separated people with children because of jurisdiction regarding parents’ rights after divorce. We conducted additional analyses exploring this possibility and describe our findings in the Results section.

5 Variables with missing values (and % missing) include homeownership (0.003%), age (0.001%), race/ethnicity (3.3%), total family income (0.5%), years of education (2%), employment status (0.5%), size of the largest city in the county (0.2%), difference in age from ex-partner (0.05%) and difference in education from ex-partner (5.7%). We use multiple imputation with chained equations to predict missing values. Following White et al. (2011), we include all study variables and outcomes in the imputation model.

6 We compared all of our results to the results generated by other methods which account for non-independence of observations, including standard fixed effects and random effects logit models, as well as random effects models utilizing the Mundlak formulation (1978) to avoid omitted variable bias (Bell and Jones 2015). Our substantive findings remain consistent across the alternate modeling strategies. We opted to present the simpler logistic regressions with clustered standard errors because they enable us to estimate the effects of time-invariant characteristics, like sex, and because they ease the calculation and interpretation of marginal (population-averaged) effects (Williams 2018). Results from the alternative models are available upon request.

7 As a supplementary analysis, we also estimated period effects by interacting time period with employment status, marital status, family living close, and gender. Only gender demonstrated a significant interaction with time period. Specifically, we found that women were more likely to migrate in the later years (2001–2013) than in the earlier years (1983–1989). However, this finding applies to women in general and not separated women specifically, as models for separated people do not reveal statistically significant interactions between gender and time period.

8 We also performed analyses of moving to a location close to family but ran into small-N problems.

9 We again performed supplementary analysis interacting gender and time period. Results indicated that women were more likely to onward migrate (versus not migrating) in the later years (2001–2013) compared to the earlier years (1983–1989). But, they were not more likely to engage in return migration. In other words, the interaction between gender and time period is not statistically significant for return migration but it is statistically significant for migrating elsewhere. This finding applies to women in general and not separated women specifically, as multinomial models for separated people do not find significant interactions between gender and time period.

## Appendix

**Table A.1:** Logistic regression of migration (ref. did not migrate)

	OR	SE
Marital status (ref = separated)		
Married	.468***	.035
Never-married	.489***	.039
Migrated before	2.263***	.079
Female	.882***	.022
Non-resident parent within 50 km	.515***	.050
Non-resident sibling within 50 km	.515***	.048
Non-resident child within 50 km	.449***	.054
No non-resident kin in PSID data	.646***	.043
Parents living together	1.124**	.046
Resident parents or siblings	.748***	.045
Resident child < 18	.533***	.048
Resident child 18+	.656***	.044
Age	.962***	.002
Race (ref = non-Latino white)		
Non-Latino black	.578***	.033
Other	.526***	.050
Homeowner	.345***	.014
Total family income (logged)	.956**	.016
Education in years	1.083***	.010
Employment (ref = employed)		
Unemployed	1.541***	.074
Not in labor force	1.820***	.071
Region (ref = Northeast)		
Midwest	1.223**	.088
South	1.420***	.092
West	1.674***	.122
Size of largest city in county (ref=<25,000)		
25,000–99,999	1.061	.050
100,000 or more	.839***	.038
Year		
1990 to 1999	1.044	.045
2001 to 2013	.951	.073
Study interval length	1.834***	.121
Interactions		
Married * parents within 50 km	1.055	.122
Never-married * parents within 50 km	1.046	.123
Married * siblings within 50 km	1.346**	.149
Never-married * siblings within 50 km	1.120	.127
Married * child within 50 km	1.260	.175
Never-married * child within 50 km	1.664*	.398
Married * resident child <18	1.111	.110
Never-married * resident child <18	1.268	.159
Constant	.119***	.022
N (person-intervals)	181,628	

* p < .05,

** p < .01,

*** p < .001.

Note: Pooled results of 10 imputation datasets; standard errors are clustered by family identification number.

**Table A.2:** Multinomial logistic regression of migration type for those who migrated before (ref. did not migrate) (N = 100,400)

	Returned to county where grew up		Migrated elsewhere	
	RRR	SE	RRR	SE
Marital status (ref = separated)				
Married	.359***	.059	.528***	.049
Never-married	.424***	.080	.504***	.053
Female	.834**	.054	.881***	.028
Parent in county where grew up	4.609***	.850	.632**	.105
Sibling in county where grew up	1.068	.194	1.149	.184
Child in county where grew up	3.020**	1.044	1.144	.294
Non-resident parent within 50 km	.204***	.033	.564***	.036
Non-resident sibling within 50 km	.397***	.049	.575***	.035
Non-resident child within 50 km	.529***	.078	.490***	.039
No non-resident kin in PSID data	1.082	.175	.662***	.055
Parents living together	1.005	.081	1.063	.061
Resident parents or siblings	.811	.112	.741**	.073
Resident child < 18	.770	.128	.571***	.067
Resident child 18+	.811	.136	.651***	.051
Age	.941***	.006	.969***	.002
Race				
Non-Latino black	.821*	.077	.530***	.038
Other	.474***	.080	.507***	.058
Homeowner	.310***	.026	.334***	.017
Total family income	.904***	.024	.980	.023
Education in years	.979	.014	1.088***	.012
Employment				
Unemployed	1.817***	.181	1.431***	.097
Not in labor force	1.914***	.162	1.798***	.089
Size of largest city in county				
25,000–99,999	1.089	.100	1.023	.057
100,000 or more	.781**	.066	.838**	.045
Region				
Midwest	1.152	.146	1.171	.103
South	1.556***	.175	1.406***	.112
West	1.583***	.199	1.599***	.143
Year				
1990 to 1999	.972	.084	1.118*	.060
2001 to 2013	1.024	.178	.925	.087
Study interval length	1.860***	.288	1.917***	.153
Interactions				
Married * parents in county where grew up	.412***	.089	1.670**	.278
Never-married * parents in county where grew up	.540**	.127	1.270	.242
Married * siblings in county where grew up	1.554*	.334	.855	.146
Never-married * siblings in county where grew up	1.462	.333	.875	.167
Married * child in county where grew up	1.722	.688	1.044	.336
Never-married * child in county where grew up	1.105	.515	1.420	.740
Married * resident child <18	.953	.184	1.114	.141
Never-married * resident child <18	1.455	.334	1.325	.252
Constant	.395*	.154	.128***	.030
N (person-intervals)	1,411		5,001	

* p < .05,

** p < .01,

*** p < .001.

Note: Pooled results of 10 imputation datasets; standard errors are clustered by family identification number.

**Table A.3:** Logistic regression of migration (ref. did not migrate) for separated people only

	OR	SE
Migrated before (lives outside of county of birth)	2.726***	.241
Female	.896	.129
Non-resident parent within 50 km	.450***	.069
Non-resident sibling within 50 km	.578***	.080
Non-resident child within 50 km	.357***	.055
No non-resident kin in PSID data	.394*	.151
Parents living together	1.066	.109
Resident parents or siblings	.562***	.082
Resident child < 18	.384***	.074
Resident child 18+	.671**	.100
Age	.965***	.005
Race (ref = non-Latino white)		
Non-Latino black	.598***	.068
Other	.675	.143
Homeowner	.470***	.040
Total family income (logged)	1.024	.048
Education in years	1.015	.022
Employment (ref = employed)		
Unemployed	2.078***	.241
Not in labor force	1.895***	.204
Region (ref = Northeast)		
Midwest	1.588**	.281
South	1.759**	.289
West	2.012***	.344
Size of largest city in county (ref=<25,000)		
25,000–99,999	.909	.095
100,000 or more	.662***	.065
Year		
1990 to 1999	1.106	.116
2001 to 2013	1.170	.226
Study interval length	1.466*	.239
Years since separation	.858***	.018
Difference in age with ex-partner	1.008	.008
Difference in education with ex-partner	.983	.021
Ratio of income with ex-partner	1.063	.162
Interactions		
Female * parents within 50 km	1.100	.227
Female * siblings within 50 km	.962	.182
Female * child within 50 km	1.459	.327
Female * resident child <18	1.418	.308
Constant	.244***	.097
N (person-intervals)	12,713	

* p < .05,

** p < .01,

*** p < .001.

Note: Pooled results of 10 imputation datasets; standard errors are clustered by family identification number.

**Table A.4:** Multinomial logistic regression of migration type for those who migrated before for separated people only (ref. did not migrate) (N = 6,218)

	Returned to county where grew up		Migrated elsewhere	
	RRR	SE	RRR	SE
Female	.590	.177	.828	.139
Parent in county of birth	3.770***	1.030	.498**	.115
Sibling in county of birth	1.220	.354	.997	.222
Child in county of birth	1.490	.645	.947	.328
Non-resident parent within 50 km	.153***	.047	.523***	.084
Non-resident sibling within 50 km	.424***	.097	.557***	.087
Non-resident child within 50 km	.526**	.118	.407***	.062
No non-resident kin in PSID data	.233	.242	.549	.260
Parents living together	.899	.163	1.037	.153
Resident parents or siblings	.676	.240	.540*	.136
Resident child < 18	.539	.197	.388***	.102
Resident child 18+	1.294	.354	.610**	.110
Age	.948***	.010	.972***	.006
Race				
Non-Latino black	.725	.145	.522***	.084
Other	.366**	.137	.809	.240
Homeowner	.434***	.076	.487***	.056
Total family income	.966	.077	1.107	.075
Education in years	.912*	.034	1.034	.029
Employment				
Unemployed	2.463***	.557	2.331***	.376
Not in labor force	2.329***	.453	2.038***	.316
Size of largest city in county				
25,000–99,999	1.093	.206	.813	.114
100,000 or more	.711	.131	.605***	.083
Region				
Midwest	1.788	.547	1.443	.352
South	2.057*	.593	1.509	.351
West	1.777	.568	1.884**	.438
Year				
1990 to 1999	1.043	.205	1.007	.146
2001 to 2013	.727	.268	1.169	.320
Study interval length	2.297**	.692	1.319	.317
Years since separation	.780***	.036	.876***	.025
Difference in age with ex-partner	1.038*	.015	1.005	.009
Difference in education with ex-partner	1.019	.042	.946	.028
Ratio of income with ex-partner	.738	.205	1.055	.211
Interactions				
Female * parents in county of birth	1.587	.616	1.451	.469
Female * siblings in county of birth	.930	.365	1.432	.420
Female * child in county of birth	4.416*	2.717	1.955	1.020
Female * resident child <18	1.585	.681	1.345	.389
Constant	.743	.552	.308*	.162
N (person-intervals)	259		537	

* p < .05,

** p < .01,

*** p < .001.

Note: Pooled results of 10 imputation datasets; standard errors are clustered by family identification number.
